# Supplementary figures and images for: The Lyme disease bacterium, Borrelia burgdorferi, stimulates an inflammatory response in human choroid plexus epithelial cells
Source: PLoS One. 2020 Jul 9;15(7):e0234993. doi: 10.1371/journal.pone.0234993 (PMC7347220; doi:10.1371/journal.pone.0234993)

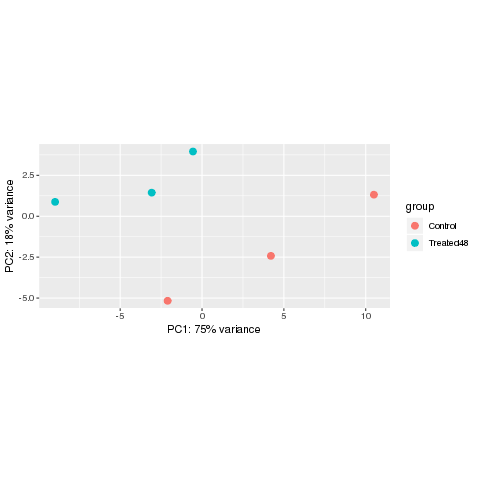

Supplement: S1 Fig — (PNG) [file pone.0234993.s003.png]
