# Supplementary material for: The Lyme disease bacterium, Borrelia burgdorferi, stimulates an inflammatory response in human choroid plexus epithelial cells
Source: PLoS One. 2020 Jul 9;15(7):e0234993. doi: 10.1371/journal.pone.0234993 (PMC7347220; doi:10.1371/journal.pone.0234993)
Supplement: S1 Raw Images — (PDF) [file pone.0234993.s004.pdf]

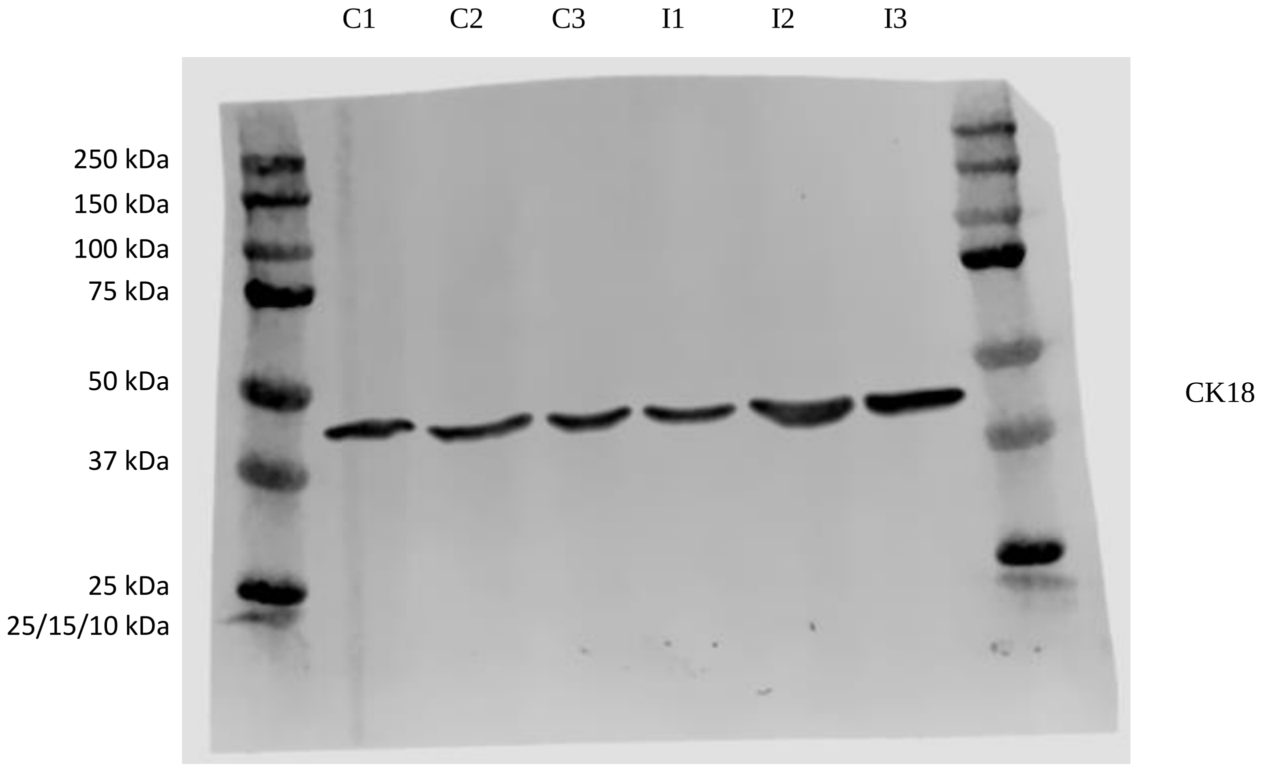

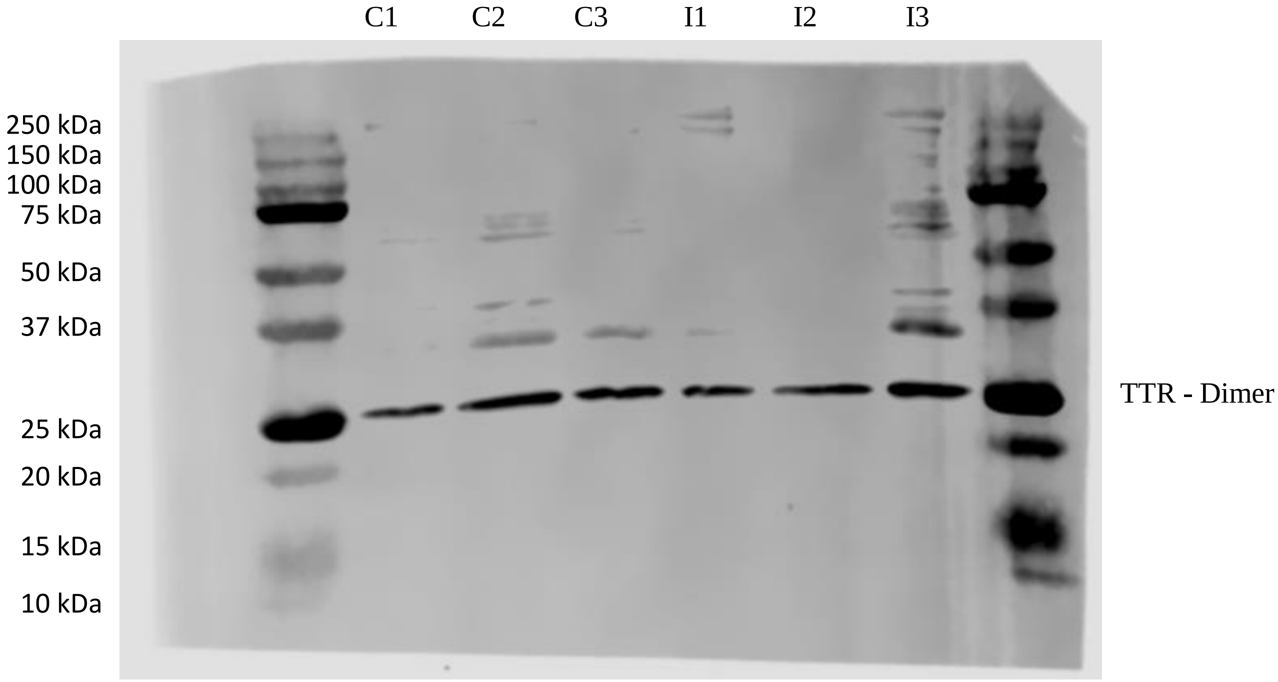

Fig1C. Protein lysates were isolated from control (C1, C2, C3) and infected groups (I1, I2, I3) N=3. Western Blots for CK18 and TTR were ran on separate gels/blots as shown. The goal for these blots was to determine the presence of these choroid plexus markers within our samples and as such no quantification was performed.
